# Non-*C. albicans Candida* Species Develop Clinically Relevant Biofilms on Stainless Steel Under Respiratory Tract-Mimicking Conditions

**DOI:** 10.3390/life16010148

**Published:** 2026-01-16

**Authors:** Natalia Bagon, Vlaudimir Marques, Deisiany Ferreira, Melyssa Negri

**Affiliations:** 1 Postgraduate Program in Health Sciences, Universidade Estadual de Maringá, Maringá 87020-900, Brazil; na.pecin_@hotmail.com (N.B.); deisygomes30@gmail.com (D.F.); 2 Maringa Regional University Hospital, Universidade Estadual de Maringá, Maringá 87020-900, Brazil; vdmarques@uem.br; 3 Medical Mycology Laboratory, Department of Clinical Analysis, Universidade Estadual de Maringá, Maringá 87020-900, Brazil

**Keywords:** fungal biofilm, SEM, artificial saliva, metal cannula, *Candida* spp.

## Abstract

Biofilm formation by non-*C. albicans Candida* (NAC) species is a major factor in device-associated infections, yet few studies have examined their development under physiologically relevant conditions. This study evaluated the biofilm-forming capacity of *Candida tropicalis*, *Candida parapsilosis sensu stricto* and *Candida albicans* on stainless steel surfaces in the presence of artificial saliva, simulating the respiratory tract environment of tracheostomized patients. Standardized inocula were incubated for 24 h, and biofilms were assessed through quantification of viable cells, biomass, biofilm matrix production and structural characterization by scanning electron microscopy (SEM). *C. tropicalis* produced the most robust biofilms compared to *C. albicans* and *C. parapsilosis* stricto sensu isolates, with significantly higher biomass and biofilm matrix (*p* < 0.001). *C. parapsilosis sensu stricto* developed less dense yet structurally defined biofilm networks. SEM confirmed mature and compact biofilm architecture, especially in *C. tropicalis*. These results demonstrate the strong intrinsic biofilm-forming ability of NAC species on stainless steel under host-like conditions, reinforcing their capacity to persist on medical surfaces and their relevance as independent contributors to biofilm-related contamination and infection.

## 1. Introduction

Yeasts of the genus *Candida* have a remarkable ability to adhere to surfaces and develop structured biofilms, contributing to their persistence in both the host and abiotic environments. Although *Candida albicans* is the most extensively characterized species, non-*C. albicans Candida* (NAC) species, such as *Candida parapsilosis sensu stricto* and *Candida tropicalis*, have received increasing attention due to the rising incidence rates, mainly in Intensive Care Units (ICUs) [1]. In the United States, *Candida* spp. is the fourth leading cause of nosocomial bloodstream infections, with mortality rates exceeding 40% [2]. Globally, *C. tropicalis* has emerged as a significant pathogen, particularly in tropical regions, where it ranks among the leading causes of candidemia with emerging profiles of antifungal resistance [3,4,5,6,7]. Multicenter studies (2020 to 2024) reinforce this trend, showing that *C. tropicalis* and *C. parapsilosis* each account for approximately 17.2% of isolates, highlighting the need for continuous surveillance and targeted prevention strategies in hospital settings [8,9,10].

The biological relevance of NAC species is underscored by their distinct adhesion mechanisms, adaptive responses, and ability to form robust biofilms with specific architectures [11,12,13,14]. This process is influenced by the physicochemical properties of the substrate and the surrounding environment [15]. Salivary environments, whether natural or simulated, contain mucins and ions capable of conditioning solid surfaces and modulating microbial adhesion [16,17]. Experimental studies show that the formation of a salivary film alters surface free energy and cell surface hydrophobicity, significantly affecting the biofilm behavior of NAC species depending on the species and biomaterial involved [16,17,18]. In clinical settings, these host–pathogen interactions are further influenced by medical devices (catheters, probes, and cannulas) that provide new abiotic surfaces for colonization, often facilitated by host immunosuppression [19].

Individuals on mechanical ventilation are particularly predisposed to colonization, as endotracheal tubes (ETTs) provide surfaces conducive to adhesion and induce physiological dysfunction in the respiratory tract [20,21]. These devices compromise mucociliary clearance and promote the accumulation of secretions, favoring fungal proliferation [16,17]. Due to the damage associated with long-term use of ETTs, tracheostomy becomes a viable option for patients who require continuous airway support [22,23]. This invasive procedure involves an opening in the trachea maintained by a metal or non-metal cannula [24]. Despite being easy to clean, metallic tracheostomy tubes are susceptible to colonization by the patient’s microbiota, providing a surface for fungal biofilm formation and subsequent infection [25].

Stainless steel is widely used in medical devices due to its hydrophilic and chemically stable surface, which directly influences microbial adhesion and biofilm architecture [18,25]. In this context, the interaction between *Candida* spp. and metal surfaces under salivary conditions becomes crucial, as salivary components modify surface topography and fungal colonization, allowing abiotic surfaces to act as niches for silent persistence before clinical manifestation [15,16,17,18]. Given that 60% to 70% of nosocomial infections are associated with such devices, where colonization frequently precedes recurrent infectious processes, understanding the intrinsic biofilm-forming capacity of NAC species under host-mimicking conditions is vital [1,19,25]. Therefore, investigating the factors that modulate this adhesion is essential to identifying critical points and developing more effective control strategies and preventive measures in clinical settings [1,15,25].

Considering the limited research addressing NAC biofilm development on metallic devices in the presence of saliva-like fluids, this research sought to investigate the biofilm formation of *Candida* yeasts on stainless steel in the presence of artificial saliva, as well as their susceptibility to main antifungal agents. This model provides a controlled and physiologically relevant environment to explore fungal adhesion, maturation, matrix production, and biomass production. Ultimately, this study aims to clarify the antifungal resistance profile and the virulence of these species under conditions that simulate the respiratory tract environment.

## 2. Materials and Methods

### 2.1. Isolation Site and Identification of the Yeasts

For yeasts isolation, samples were collected from tracheostomized patients after bronchoscopic examination. The isolates were collected from tracheal aspirate, tracheal biopsy and metal cannula. The tracheal aspirate was collected under vacuum in a dry, sterile container; the tracheal biopsy fragment was collected with sterile forceps from the mucosa of the tracheal wall and placed in a conical tube with phosphate-buffered saline (PBS), pH 7.4; and both the inner and outer parts of the metal cannula were collected and stored in a sterile glass container. In the laboratory, the tracheal aspirate was centrifuged at 3500× *g* rpm for 5 min, and the supernatant was discarded, while the tracheal biopsy fragment was cut into smaller pieces in a sterile Petri dish. Both the inner and outer surfaces of the metal cannula parts were scraped with a cytological brush, which was then sonicated for 50 s, at an amplitude of 30%, in PBS, to detach potential biofilm and dissociate the cells. The sonicated sample was then centrifuged, the supernatant discarded, and the precipitate was resuspended in PBS.

After processing, all samples were seeded on Sabouraud dextrose agar (SDA; Kasvi, Madrid, Spain) plus 0.02% chloramphenicol and incubated at 25 °C for a maximum period of 15 days. All fungi that were isolated from the respiratory samples exhibited growth greater than or equal to 1.5 × 10^4^ colony forming units (CFU/mL). Plates with growth were screened by culturing the fungi in CHROMagar™ *Candida* (BD Difco™, Franklin Lakes, NJ, USA) and identified by morphological and biochemical characteristics, as described by De Hoog et al. [26] and Westblade et al. [27]. Species were confirmed using matrix-assisted laser desorption ionization-fingerprinting time-of-flight mass spectrometry (MALDI-TOF MS) according to Pascon et al. [28], with the readings and interpretation performed on a VITEK^®^ MS with MYLA^®^ V3.0 software (bioMérieux, Marcy l’Etoile, France).

The isolates were identified and subsequently deposited in the Microbiological Collections of the Paraná Network—TAXonline (CMRP) at the Federal University of Paraná and in the Teaching and Research Laboratory in Clinical Analysis (LEPAC), Medical Mycology Laboratory of the State University of Maringá, under the codes CMRP5750, CMRP5751, CMRP5752 (*C. albicans*); CMRP5745 and CMRP5749 (*C. parapsilosis sensu stricto*); CMRP5746, CMRP5747, CMRP5748 (*C. tropicalis*). The study was approved by the Permanent Ethics Committee on Research involving Human Beings of the State University of Maringá, number 49603521.2.0000.0104 and this study was registered in the National System for the Management of Genetic Heritage and Associated Traditional Knowledge (Sistema Nacional de Gestão do Patrimônio Genético e do Conhecimento Tradicional Associado—SisGen) under the code AB1125A.

### 2.2. Minimum Inhibitory Concentration (MIC) Determination

The MIC of isolates was determined using the micro broth dilution technique with amphotericin B (Sigma-Aldrich, St. Louis, MO, USA), fluconazole (Laboratório Sanobiol Ltd.a, Pouso Alegre, Minas Gerais, Brazil), and nystatin (Sigma-Aldrich, St. Louis, MO, USA). An American Type Culture Collection (ATCC) reference strain of *C. albicans* (ATCC 90028) was included as an assay control. Concentration series of the drugs were prepared at a 16 μg/mL to 0.03 μg/mL for amphotericin B and nystatin, and 64 μg/mL to 0.125 μg/mL for fluconazole, in Roswell Park Memorial Institute 1640 medium, with L-glutamine, 2% glucose (RPMI 1640; Sigma-Aldrich, St. Louis, MO, USA) and 0.165 M 3-(N-morpholino) propanesulfonic acid buffer (MOPS; Sigma-Aldrich, St. Louis, MO, USA), pH 7.05.

Wells of a 96-well plate containing the drugs were inoculated with the yeast at a final concentration of 5 × 10^3^ CFU/mL. After incubation at 37 °C for 48 h, the reading for assays involving amphotericin B and fluconazole was performed in a microplate reader at 530 nm (SpectraMax^®^ Plus 384 Microplate Reader, Molecular Devices, San Jose, CA, USA), while for the nystatin assays, a visual reading was performed. The MIC was defined as the lowest concentration of the antifungal capable of inhibiting 50% of the cell growth for fluconazole or 90% of the cell growth for amphotericin B and nystatin in concerning the respective control without drug. The interpretation of the cutoff points was in accordance with CLSI M27-A2 for fluconazole and amphotericin B, while for nystatin, the yeast were considered susceptible when the MIC was ≤4 μg/mL [29,30,31].

### 2.3. Biofilm Formation Respiratory Tract-Mimicking Conditions

To simulate the respiratory environment from which the yeasts were isolated and evaluate their ability to form biofilm, the tests were conducted on stainless steel coupons (1.6 cm^2^; 410 stainless steel; Aperam, Timóteo, Minas Gerais, Brazil) in the presence of artificial saliva. Briefly, the isolates were inoculated in Sabouraud dextrose broth (SDB; Acumedia Manufacturers, Lansing, MI, USA) and incubated for 18 h at 37 °C under shaking at 130 rpm. After incubation, cells were centrifuged at 8000× *g* rpm for 5 min at 4 °C and washed twice with PBS, then adjusted to a concentration of 1 × 10^7^ CFU/mL in a solution (1:1) of PBS and artificial saliva (Botica Ouro Preto, Maringá, Paraná, Brazil). The ability of the isolates to form biofilm on stainless steel was performed according to the protocol of Queiroz et al. [31], with adaptations. The artificial saliva used was composed of carboxymethylcellulose (10 g/L), sorbitol (24 g/L), potassium chloride (0.96 g/L), sodium chloride (0.67 g/L), magnesium chloride (0.04 g/L), calcium chloride (0.12 g/L), potassium phosphate (0.27 g/L), nipagin (2 g/L), nipazol (1 g/L) and sterile purified water. In each well of a 24-well polystyrene plate a test specimen of stainless steel coupon, previously sterilized, was placed with 500 μL of the inoculum. The plate was incubated at 37 °C under shaking at 110 rpm. After the adhesion step (2 h), the medium was aspirated to remove non-adherent yeast, washed once with PBS and fresh PBS and artificial saliva solution (1:1 *v*/*v*) was added. After 24 h, the wells were washed three times with PBS and each steel specimen was transferred to a new 24-well plate for the respective tests. Assays were performed in quadruplicate.

### 2.4. Biofilm Analysis: Total Biofilm Biomass, Matrix Analysis and Cell Viability

Total biomass and biofilm matrix were quantified by the colorimetric crystal violet (CV) staining method and safranin staining, respectively. The biofilm on the steel plate specimens was fixed with 500 μL of 100% methanol (Anidrol, Diadema, São Paulo, Brazil) in 24-well plates for 15 min. After removing the methanol, the plates were dried at room temperature and 500 μL of 1% CV was added for 5 min, protected from light. The wells were washed twice with sterile distilled water, then the steel plate specimens were transferred to new wells, and 500 μL of 33% acetic acid was added to dissolve the dye. The absorbance was read at 530 nm. The average absorbance of CV retained by attached or biofilm yeast was expressed as absorbance per unit area of the stainless steel plate (abs530/cm^2^). Assays were performed in duplicate, following the method described by reference [32]. Biofilm matrix quantification was performed by safranin staining according to Seidler et al. [33], with adaptations. The biofilm on the steel plate specimens were stained with 500 μL of 1% safranin (Vetec Química Fina, Duque de Caxias, Rio de Janeiro, Brazil) in 24-well plates, protected from light. After 5 min, the wells were carefully washed with sterile distilled water and the steel plate specimens were transferred to new wells. The specimens were bleached with 500 μL of 33% acetic acid solution and the absorbance was read at 492 nm. The matrix analysis tests were performed in quadruplicate and the results are standardized per unit area of the stainless steel plate (abs492/cm^2^).

To determine cell viability, each steel specimen was scraped vigorously and the contents were transferred to a conical tube. The samples were sonicated at 30% amplitude for 50 s to detach the biofilm then centrifuged at 8000× *g* rpm for 5 min and resuspended in PBS. Serial dilutions were performed and each dilution was seeded in SDA supplemented 0.02% chloramphenicol then incubated at 37 °C. The number of viable and culturable cells was determined after 48 h and the total CFU per unit area (log10 CFU/cm^2^) of the stainless steel plate was enumerated.

### 2.5. Scanning Electron Microscopy (SEM)

To analyze the biofilm structure, biofilm on the steel plate specimens was fixed with 2 mL of 0.1 M sodium cacodylate solution (Sigma-Aldrich, St. Louis, MO, USA) and 2.5% glutaraldehyde (EMS, Hatfield, PA, USA). The specimens were dehydrated with alcohol, using 50% to 100% ethanol, for a submersion period of 15 min. Before observation, the steel plate specimens were mounted on stubs, metallized with gold, and observed by SEM (Quanta™ 250; FEI Company, Hillsboro, OR, USA). Images were taken at 5000× magnification.

### 2.6. Statistical Analysis

Data are expressed as means ± standard deviation (SD). Significant differences between means were identified using the one-way ANOVA test, followed by the Bonferroni multiple comparison test, or Student’s *t* test, followed by the Mann–Whitney U test. Data were analyzed using Prism 6 software (GraphPad, San Diego, CA, USA). Values of *p* < 0.05 were considered statistically significant.

## 3. Results

In total, eight isolates of the genus *Candida* were found in the samples analyzed (tracheal aspirate, biopsy and metal cannula), five of which were NAC species and three were identified as *C. albicans* (Table 1).

When evaluating the susceptibility profile to conventional antifungals, all *Candida* spp. isolates were susceptible to the drugs amphotericin B, fluconazole, and nystatin (Table 2).

**Figure 1:**
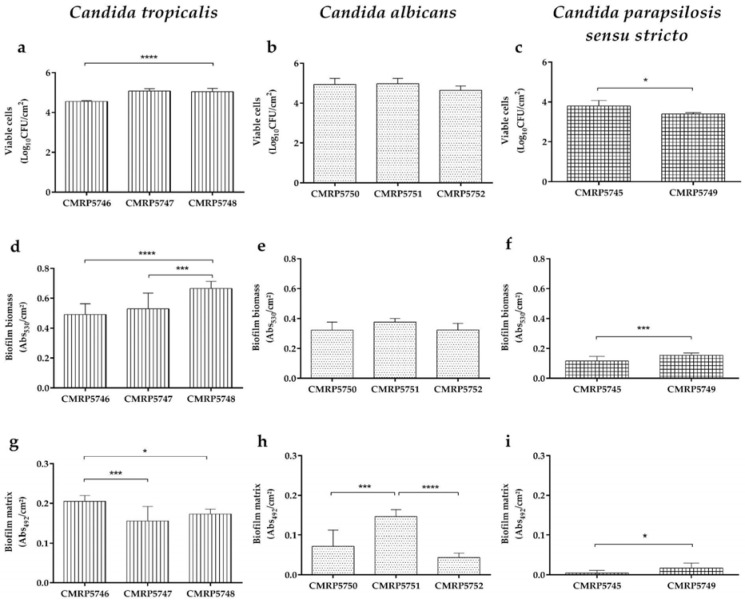
(**a**–**i**) In vitro analysis of biofilm formation in 24 h on stainless steel and artificial saliva of isolates (**a**,**d**,**g**) *C. tropicalis*; (**b**,**e**,**h**) *C. albicans*; (**c**,**f**,**i**) *C. parapsilosis sensu stricto*; in terms of (**a**,**b**,**c**) quantifying viable and culturable cells (log10 CFU/cm^2^); (**d**,**e**,**f**) total biomass (abs530/cm^2^); (**g**,**h**,**i**) biofilm matrix content (abs492/cm^2^). Indicates a statistically significant difference * *p* < 0.05, *** *p* < 0.001, **** *p* < 0.0001.

**Figure 2:**
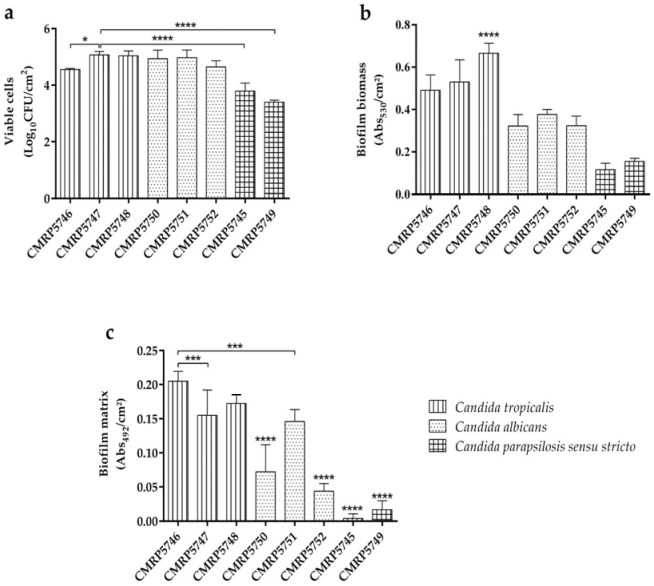
Comparing all isolates in respiratory samples in terms of (**a**) viable and culturable cells (log10 CFU/cm^2^); (**b**) biofilm biomass (abs530/cm^2^); (**c**) and biofilm matrix (abs492/cm^2^). Significant differences between data are denoted by * for *p* < 0.05, *** for *p* < 0.001 and **** for *p* < 0.0001.

All clinical isolates were capable of forming biofilm within 24 h on stainless steel in the presence of artificial saliva (Figure 1). The biofilm formation capacity differed significantly (*p* < 0.05) among the species evaluated: *C. tropicalis* (CMRP5748, CMRP5746, CMRP5747), *C. albicans* (CMRP5751, CMRP5750, and CMRP5752), and *C. parapsilosis sensu stricto* (CMRP5745 and CMRP5749), across the analyzed parameters (Figure 2).

**Figure 3:**
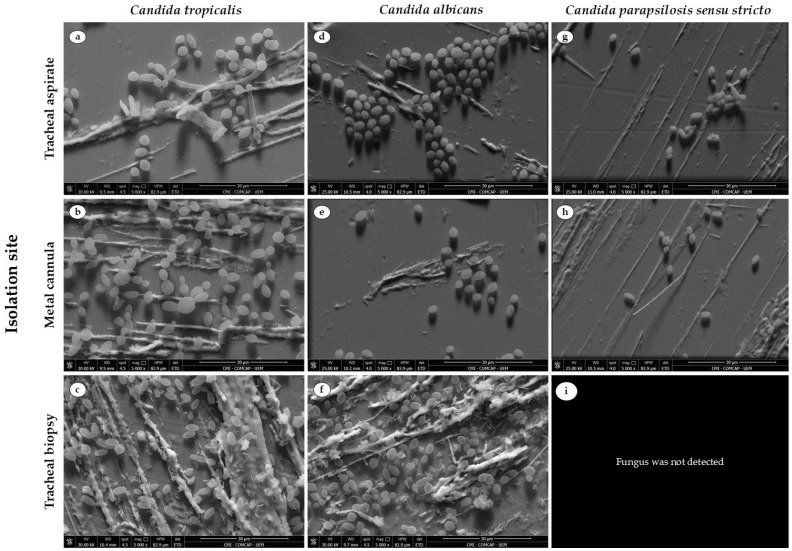
Scanning electron microscopy (SEM) images of 24 h biofilms grown on stainless steel coupons in artificial saliva: (**a**) *C. tropicalis* CMRP5746, (**b**) *C. tropicalis* CMRP5748, (**c**) *C. tropicalis* CMRP5747; (**d**) *C. albicans* CMRP5750, (**e**) *C. albicans* CMRP5752, (**f**) *C. albicans* CMRP5751; (**g**) *C. parapsilosis sensu stricto* CMRP5745, (**h**) *C. parapsilosis sensu stricto* CMRP5749, (**i**) No fungi was detected in this site. Isolates were collected from tracheal aspirate (**a**,**d**,**g**), tracheal biopsy (**c**,**f**), and metal tracheostomy cannula (**b**,**e**,**h**). Images obtained at 5000× magnification. Scale bar: 30 μm.

*C. tropicalis* demonstrated a superior biofilm-forming capacity on stainless steel, exhibiting greater total biomass production compared to the other isolates, regardless of the isolation site (*p* < 0.0001; Figure 2b). The isolate CMRP5748 (from metal cannula) showed the highest performance with 0.664 ± 0.05 abs530/cm^2^ of total biomass (*p* < 0.001; Figure 1d). This species was also prominent for its greater production of biofilm matrix (Figure 1g and Figure 2c), with high concentrations observed in isolates from tracheal aspirate (0.204 ± 0.01 abs492/cm^2^ for CMRP5746; Figure 2b) and those from the metal cannula (0.172 ± 0.01 abs492/cm^2^ for CMRP5748). Additionally, the isolates maintained a high viable cell count, particularly the isolate CMRP5747 (from tracheal biopsy), reaching 5.072 ± 0.12 log10 CFU/cm^2^ (Figure 1a and Figure 2a). SEM analysis corroborated the quantitative findings, revealing that the three *C. tropicalis* isolates were capable of producing biofilms. However, particular attention is drawn to the isolate from the tracheal biopsy, which yielded the most structured and dense biofilm in comparison to the other isolates (Figure 3a–c).

The *C. albicans* isolates demonstrated an intermediate biofilm-forming capacity compared to the isolates of other species. The isolates CMRP5751 (from tracheal biopsy) and CMRP5750 (from tracheal aspirate) showed high viable cell counts, 4.977 ± 0.27 and 4.939 ± 0.30 log10 CFU/cm^2^ respectively (Figure 1b). Total biomass (Figure 1e) ranged from 0.322 ± 0.05 abs530/cm^2^ (CMRP5750) to 0.377 ± 0.02 abs530/cm^2^ (CMRP5751). Regarding the biofilm matrix (Figure 1h), production was lower compared to the *C. tropicalis* isolates, reaching a maximum of 0.146 ± 0.02 abs492/cm^2^ in isolate CMRP5751 (from tracheal biopsy) (*p* < 0.001). SEM images (Figure 3d–f) revealed less structured biofilms than those of *C. tropicalis*, but confirmed the presence of viable cells and matrix, consistent with the quantitative findings. Interestingly, SEM evidenced that, similar to the *C. tropicalis* isolate, the isolate CMRP5751 from the tracheal biopsy presented a denser and more structured biofilm than the isolates from other sites.

Regarding the analysis of *C. parapsilosis sensu stricto* isolates, it is important to emphasize that the presence of yeasts of this species was only identified in the tracheal aspirate and metal cannula and was not identified in the tracheal biopsy. These isolates demonstrated the lowest biofilm-forming capacity among the evaluated isolates. This low capacity was evidenced by the lowest viable cell counts, varying between 3.396 ± 0.07 and 3.794 ± 0.28 log10 CFU/cm^2^ for isolates CMRP5749 and CMRP5745, respectively (Figure 1c). The most notable difference was the significantly reduced matrix production compared to the other isolates (*p* < 0.05, Figure 2c); the CMRP5745 isolate (tracheal aspirate) presented only 0.004 ± 0.01 abs492/cm^2^ and the CMRP5749 isolate (metal cannula) presented 0.016 ± 0.01 abs492/cm^2^ (Figure 1i and Figure 2c). These results indicate a reduced efficiency in the formation of structured biofilms on stainless steel in the presence of artificial saliva. SEM images (Figure 3g,h) confirmed this lower capacity, evidencing a reduced number of cells and a scarce or absent biofilm matrix, especially in comparison with the *C. tropicalis* isolates (Figure 3c).

## 4. Discussion

The colonization of the respiratory tract by yeasts of the genus *Candida*, including *C. albicans* and Non-*albicans Candida* (NAC) species, represents a critical risk factor in tracheostomized patients. While these yeasts are common constituents of the human microbiota, they emerge as primary opportunistic agents in immunocompromised hosts, often leading to severe secondary infections [24]. Our findings underscore that metal tracheostomy cannulas serve as stable abiotic reservoirs for microbial attachment, significantly increasing the risk of systemic dissemination and candidemia [34,35]. This observation is corroborated by Meng et al. [8], who reported that the incidence of *C. tropicalis* bloodstream infections has been increasing annually, particularly in high-risk hospital units, where it accounts for a substantial percentage of candidemia cases with high mortality rates [8].

Despite the global trend of emerging antifungal resistance, all isolates in this study demonstrated susceptibility to fluconazole, amphotericin B, and nystatin. However, the higher MICs observed for *C. parapsilosis* sensu stricto (2.0 μg/mL to 4.0 μg/mL) warrant attention. In a broader comparison with existing literature to clarify the study’s novelty, Meng et al. [8] recently highlighted that *C. tropicalis* isolates frequently exhibit higher resistance rates to azoles compared to other species, suggesting that the susceptibility profile is highly species-dependent and niche-specific. Although our clinical isolates were susceptible, this does not diminish the risk of infection, as medical devices facilitate the transition from planktonic cells to highly resilient biofilms [34,36,37].

A major highlight of this study was the predominance and superior biofilm performance of *C. tropicalis* across all tracheal niches. This can be attributed to several species-specific factors [38]. Scientific evidence suggests that *C. tropicalis* possesses higher phenotypic plasticity and metabolic activity in host-mimicking environments compared to *C. albicans* and *C. parapsilosis* [39]. As demonstrated by De Souza et al. [39], this species triggers specific molecular adaptations when exposed to host-like conditions, such as the artificial saliva used in our model, leading to the upregulation of the transcription factor *EFG1* and adhesins like *ALS3*. These genetic pathways facilitate enhanced filamentation and cell wall remodeling, which are crucial for the establishment of the dense extracellular matrix observed in our SEM analysis [40]. Furthermore, the shorter time-to-positivity (TTP) often observed for *C. tropicalis* in clinical cultures indicates a faster natural growth rate, allowing it to outcompete other yeasts during the colonization of abiotic surfaces like the stainless steel cannula. Finally, the fact that the biopsy-derived isolate (CMRP5747) was the most developed suggests that direct contact with the host’s immune system selects for more virulent phenotypes with increased adherence and protective matrix production.

The simulation of the respiratory environment through the use of artificial saliva was a key methodological choice [41]. Our results align with the findings of de Souza et al. [39], who showed that environmental stress and host-like conditions (such as pH and nutrient availability) trigger cell wall modifications and the upregulation of key adhesins like *ALS3* and the transcription factor *EFG1* in *C. tropicalis*. This adaptive mechanism allows the yeast to form a protective shield, maintaining the infectious focus within the airway and increasing the risk of serious complications like ventilator-associated pneumonia (VAP) [42].

Interestingly, isolates obtained from tracheal biopsies, representing direct contact with host tissue, produced more structured and robust biofilms compared to those from aspirates (Figure 1, Figure 2 and Figure 3). This phenotypic distinction suggests that isolates exposed to the host’s immune system in vivo undergo a selection process for more resilient and aggressive strains. As evidenced by De Souza et al. [39], certain morphotypes of *C. tropicalis* are specifically adapted to colonize biological mucosa and abiotic devices, demonstrating a complex interaction between the isolate’s genetic background and the anatomical site of infection [43].

In contrast, *C. parapsilosis sensu stricto* showed the lowest biofilm production capacity in our model. While this species is a notorious colonizer of intravascular catheters, its lower efficiency in forming dense biofilms on stainless steel suggests that its virulence in the tracheostomy niche may be more dependent on environmental factors or its transmission via healthcare workers’ hands than on matrix production [44,45]. This distinction further clarifies the added value of our study, as it highlights how the substrate (metal vs. plastic) and the species identity dictate the architecture of the microbial community.

One of the main clinical concerns for tracheostomized patients remains the prevention of device-associated infections, which are often exacerbated by the persistent nature of fungal biofilms. As argued by Al Bataineh & Alazzam [46], the lack of intrinsic antimicrobial properties in conventional medical devices, such as the stainless steel cannulas, necessitates the application of advanced surface treatments and microfabrication techniques to disrupt microbial attachment [46]. Our data reinforce the need for constant evaluation of the microbial composition of metal cannulas and the development of preventive strategies focused on decolonizing these devices to reduce the infection associated with *Candida* spp. in immunocompromised populations.

Our study has inherent limitations, such as the focus on monospecies biofilms and the use of in vitro models, which may not fully capture the polymicrobial complexity of the tracheal niche. However, it provides a crucial baseline for understanding *Candida* spp. behavior on metal cannulas and highlights the aggressive nature of specific clinical isolates. We demonstrate that the high biomass and rapid maturation observed in *C. tropicalis* on stainless steel represent a significant clinical threat that mirrors global epidemiological shifts toward more virulent NAC species. According to Meng et al. [8], *C. tropicalis* has shown an increasing prevalence in intensive care settings, often linked to higher mortality and specific genetic lineages that favor environmental persistence [8]. Furthermore, as shown by De Souza et al. [39], the phenotypic plasticity of this species allows it to adapt rapidly to host-mimicking stresses, reinforcing the urgency of implementing stricter colonization monitoring protocols and personalized antifungal stewardship in ICUs to prevent the progression from colonization to systemic candidemia [39].

Our findings open important avenues for future clinical and biotechnological outcomes. Based on our data demonstrating the ability of non-albicans *Candida* species to form biofilms on metal cannula surfaces, future investigations should prioritize the development of bioactive or anti-adhesive coatings for metal cannulas, utilizing techniques such as UV-induced grafting or antimicrobial peptide immobilization, to specifically target the initial attachment of *C. tropicalis* [46]. Furthermore, expanding this research to include polymicrobial interaction models (*Candida*-bacteria our *Candida-Candida*) and transcriptomic analysis of biofilm-associated genes on metallic substrates will be essential to reflect the complex reality of the tracheostomized patient’s microbiota. Ultimately, longitudinal clinical studies are needed to establish whether biofilm density on the cannula can serve as a predictive biomarker for the risk of systemic candidemia.

## Figures and Tables

**Table 1:** Fungal species isolated and identified from the samples collected.

Isolation Site	Species
Tracheal aspirate	*C. tropicalis* (CMRP5746), *C. parapsilosis sensu stricto* (CMRP5745), *C. albicans* (CMRP5750)
Metal cannula	*C. tropicalis* (CMRP5748), *C. parapsilosis sensu stricto* (CMRP5749), *C. albicans* (CMRP5752)
Tracheal biopsy	*C. tropicalis* (CMRP5747)*, C. albicans* (CMRP5751)

**Table 2:** Antifungal minimum inhibitory concentration (MIC) values of 8 isolates from tracheal aspirate, tracheal biopsy and metal cannula.

		Antifungal Agent					
		Amphotericin B		Fluconazole		Nystatin	
		MIC (μg/mL)	Susceptibility	MIC (μg/mL)	Susceptibility	MIC (μg/mL)	Susceptibility
*C. albicans*	CMRP5750	0.5	S	0.25	S	2.0	S
	CMRP5751	0.5	S	0.125	S	2.0	S
	CMRP5752	0.5	S	0.25	S	4.0	S
*C. parapsilosis sensu stricto*	CMRP5745	1.0	S	4.0	S	2.0	S
	CMRP5749	0.5	S	4.0	S	2.0	S
*C. tropicalis*	CMRP5746	1.0	S	2.0	S	2.0	S
	CMRP5747	1.0	S	2.0	S	2.0	S
	CMRP5748	1.0	S	2.0	S	2.0	S

Breakpoints for antifungical resistance were determined according to CLSI (M27-A2) and for nystatin, the yeast were considered susceptible when the MIC was ≤4 μg/mL [29,30,31]. S: Susceptible.

## Data Availability

The original contributions presented in this study are included in the article. Further inquiries can be directed to the corresponding author(s).
